# Adolescent perspectives about their participation in alcohol intervention research in emergency care: A qualitative exploration using ethical principles as an analytical framework

**DOI:** 10.1371/journal.pone.0217855

**Published:** 2019-06-12

**Authors:** Ellen Lynch, Ruth McGovern, Catherine Elzerbi, Matthew Breckons, Paolo Deluca, Colin Drummond, Mohammed Fasihul Alam, Sadie Boniface, Simon Coulton, Eilish Gilvarry, Paul McArdle, Robert Patton, Ian Russell, John Strang, Eileen Kaner

**Affiliations:** 1 Institute of Health & Society, Newcastle University, United Kingdom; 2 Addictions Department, National Addiction Centre, Institute of Psychiatry, Psychology and Neuroscience, King's College London, London, United Kingdom; 3 Public Health Department, College of Health Sciences, Qatar University, Doha, Qatar; 4 Centre for Health Services Studies, University of Kent, Canterbury, Kent, United Kingdom; 5 Northumberland Tyne and Wear NHS Foundation Trust, Newcastle, United Kingdom; 6 School of Psychology, University of Surrey, Guildford, United Kingdom; 7 Swansea University Medical School, Swansea, United Kingdom; University of Auckland, NEW ZEALAND

## Abstract

**Aims:**

To explore adolescents’ experiences of consenting to, and participating in, alcohol intervention trials when attending for emergency care.

**Methods:**

In-depth semi-structured interviews with 27 adolescents (16 males; aged 14–17 years (M_age_ = 15.7)) who had taken part in one of two linked brief alcohol intervention trials based in 10 accident and emergency departments in England. Interviews were transcribed verbatim and subject to thematic analysis.

**Results:**

Research and intervention methods were generally found to be acceptable though confidentiality was important and parental presence could hinder truthful disclosures regarding alcohol use. Participants discussed the importance of being involved in research that was relevant to them and recognised alcohol consumption as a normative part of adolescence, highlighting the importance of having access to appropriate health information. Beyond this, they recognised the benefits and risks of trial participation for themselves and others with the majority showing a degree of altruism in considering longer term implications for others as well as themselves.

**Conclusions:**

Alcohol screening and intervention in emergency care is both acceptable and relevant to adolescents but acceptability is reliant on confidentiality being assured and may be inhibited by parental presence.

**Trial registration:**

ISRCTN Number: 45300218

## Introduction

Although the proportion of young people who have never tried alcohol has increased in recent years, alcohol remains the most widely used psychoactive drug in this population [1]. Adolescence is the key period for alcohol initiation with over 70% of young people having their first alcoholic drink by the age of 15 [2] and normative increases in both frequency and quantity of alcohol consumption occur from early adolescence through to early adulthood [3]. Adolescents may be especially vulnerable to the adverse effects of alcohol use [4]. Adolescent alcohol use can influence brain development and resultant cognitive, emotional and social development [5]. Research has identified associations between adolescent alcohol use and: heightened family conflict and lower educational outcomes [6, 7]; poorer physical [8] and mental health [9]; the development of alcohol use disorders [9, 10]; and disease in adulthood [11].

Forty per cent of adolescents aged 10–17 attending emergency departments in England reported drinking significant amounts of alcohol [12] yet a large proportion of hospitals in the UK do not offer alcohol support for young people [13]. Thus, emergency care is a key setting for prevention-focused alcohol intervention work with adolescents.

Screening and brief alcohol intervention is effective in reducing risky drinking in adults when delivered in healthcare settings [14, 15]. Although brief interventions have been shown to benefit younger people, most trials have been conducted in educational settings [16] with participants aged 18 or more [17] or have been conducted outside the United Kingdom (UK) [18–20]. A previous trial of BI delivered to underage drinkers in the ED setting in the United States of America demonstrated benefits of both therapist and tablet delivered BI in bringing about reductions in alcohol consumption at 3 month follow up and reduced alcohol consequences at 3 and 12 months post intervention [21]. Additional analysis of this data [22, 23] have shown that those who are younger, lived with their parents, reported lower alcohol consumption and higher levels of readiness to change at baseline are more likely to show positive responses to BI. These findings show promise for the effectiveness of BI in UK adolescents who, given the lower drinking age of 18 years are more likely to be younger and still living with their parents.

Despite long-standing calls for more work on preventing or reducing underage drinking [24], there remains little specific evidence to guide the prevention of alcohol-related harms in adolescents in the UK [25] Historically, this absence of evidence was due, at least in part to concern about the vulnerability of children and debate about the reliability of data collected from them [26]. However, developments in children’s rights [27] have led to greater focus on children contributing to decisions about their lives, care and treatment [28] and participation in research [29]. The challenges become most evident when research focuses on risk behaviours, as parental attitudes about the issues may hinder youth participation and thus undermine research validity and applicability [30]. There is a growing focus on the importance of gaining consent from children, including how best to do so [31–33]. However, there has been less attention on hearing young people’s direct views about participation in research [34] and little is known about the acceptability of alcohol screening and interventions methods to younger adolescents in healthcare settings in the UK.

The current study aimed to build on existing research by exploring the experiences of adolescents aged 14 to 17 who participated in two linked alcohol intervention trials (SIPS Junior) based in emergency care [35]. Although assessments of acceptability have received increasing focus in recent years [36] there is no clear consensus as to how acceptability should be defined and measured [36]. In this work the authors consider that to be acceptable research and intervention processes should not only be appropriate, comprehensible, effective and well received by participants but also ethical. The latter is particularly key in work with adolescents as there is debate as to the extent to which they can and should participate in treatment and also in research about treatment and care [28, 29].

We drew on the four principles of biomedical ethics—autonomy, beneficence, non-maleficence and justice [37]–as a framework within which to consider participants experiences of: consent and enrolment procedures; research design; and study interventions.

**Respect for autonomy**: respecting the decision-making capacities of autonomous persons; enabling individuals to make reasoned informed choices.
**Beneficence**: this balances the benefits of treatment against the risks and costs; the healthcare professional should act to benefit the patient.
**Non maleficence**: avoiding causing harm; the healthcare professional should not harm the patient. Though all treatment risks harm, that should be proportionate to the benefits of treatment.
**Justice**: distributing benefits, risks and costs fairly; patients in similar circumstances should be treated in a similar manner.

While a number of ethical guidelines have been developed to guide the ethical conduct of research and others for the provision care in an ethical manner this work was pragmatic in nature, based in routine practice and ultimately aimed to inform care improvement. As such, the contemporary view that the distinction between care and research can be overstated [38] especially in pragmatic healthcare research [39, 40] was adopted. Thus, the four widely accepted principles of biomedical ethics [37] were considered to provide an appropriate framework to guide the analysis and interpretation of data.

## Materials and methods

### Trial procedure

The randomised controlled trials aimed to evaluate the effectiveness and cost effectiveness of brief alcohol interventions for adolescents aged 14–17 who had attended 10 emergency departments in England; full protocol details have been published elsewhere [35].

Enrolled participants who scored less than 3 out of 12 on the Alcohol Use Disorders Identification Test: Consumption items (AUDIT-C) [41] were eligible for the low-risk trial whilst those scoring 3 or more were eligible for the high-risk trial. Within each trial, participants were randomised to one of: personalised feedback and brief face-to-face advice; or personalised feedback plus a smartphone or web-based electronic brief intervention (e-BI); or screening only (control group).

Those allocated to the screening only group were thanked for completing the baseline assessment and reminded that they would be contacted by the research team to complete follow—up in six and 12 months’ time.

Both interventions aimed to motivate and support young people to either reduce their drinking or delay the onset of drinking (as applicable based on low or high risk status).

Participants allocated to the personalised feedback and brief face-to-face advice group were provided with: feedback on screening results; information on recommended levels of alcohol consumption for young people; normative comparison; information about the risks associated with drinking; the potential benefits of reducing, ceasing or delaying the onset of alcohol use (and strategies to achieve this. This advice took approximately five minutes to deliver. Participants also received a copy of a leaflet summarising this information and providing details of sources of further support.

Participants in the personalised feedback and e-BI group were provided with access to the ‘SIPS City’ web application which was co-produced with young people. This application allows the user to navigate around a ‘city’ learning facts about alcohol, recording and gaining feedback on their own alcohol consumption and setting goals. Participants were provided with a demonstration of the application either on their own phone or on the tablet used for baseline data collection.

Participants in both the intervention conditions were thanked for taking part and reminded that a researcher would contact them to conduct the trial follow up in 6 and 12 months’ time.

All participants were followed up at 6 and 12 months after randomisation. Participants received a £5 gift voucher for completing the screening and baseline assessment and each follow up questionnaire. On completing 12 month follow up, participants were also entered into a prize draw to win an iPad.

### Qualitative study procedure

Between March and November 2015 data were gathered through embedded qualitative interviews which explored young peoples’ perceptions of participating in the trials after completion of the 12 months of follow-up. While interviews were scheduled to take place approximately 2 weeks after follow up this varied based on the number of contact attempts required and resultant period of time taken to contact each participant for follow up and interview. Each interview was conducted by one of two post-doctoral Research Associates, one male (MB) and one female (CE) both of whom had previous experience conducting qualitative interviews. At the beginning of each interview the researchers briefly introduced themselves giving their name and role on the project as well as the name of the institution where they were based. Both research associates were also involved in recruiting participants to the overall trials, to minimise any bias participants were never interviewed by the same researcher who recruited them.

### Participants

The pool of potential participants included all those who had consented to take part in linked trials and who agreed to be contacted about participation in an interview. Purposive sampling was based on data regarding participants’ characteristics that were collected in the parent trials. Sampling aimed to achieve a maximum variation sample based on the following criteria: age, gender, ethnicity, hospital from which they were recruited, high or low risk status, allocated intervention group, and whether a parent was present at screening. Young people in the e-BI intervention group were further sampled according to whether or not they had downloaded the intervention app. Sampled participants were posted a study information sheet along with a letter inviting them to take part in an interview. For those under 16, a parent or guardian was also sent a letter telling them about the invitation. Follow-up phone calls, conducted on at most four separate occasions, were utilised to confirm willingness to participate. Consenting participants were offered the choice between an interview face to face or by telephone; all chose telephone interviews which have been shown to generate data similar to that collected in face-to-face interviews [42]. Interviews were arranged to take place at a time convenient for the young person and when they would be comfortable talking however it was left up to the young person to decide whether they wanted to be somewhere private at the time of the telephone interview. Attempts were made to contact 139 participants in total. Of the 139 contacted, 27 agreed to be interviewed. Among the remaining 112: 11 declined to take part; 2 parents declined on participants behalf; 6 agreed to telephone interview but then failed to answer, 12 were contactable but provided no definitive agreement to participate, 5 hung up following introduction, 5 contact details were no longer active, 71 contact details appeared active but voicemail, text and/or SMS contacts were not responded to. All interviewees received a £5 gift voucher.

The final sample comprised 11 females and 16 males aged 14–17 years (M_age_ = 15.7, standard deviation [SD] = 1.30). Fifteen were higher risk drinkers (M_AUDIT C score_ = 5.6, SD = 0.70) and 12 low risk drinkers (M_AUDIT C score_ = 0.67, SD = 0.26) at baseline assessment. Participants were predominantly white (22 White, 2 Asian, 1 black, 1 mixed, 1 other). Twelve received the brief intervention face to face, 8 received the electronic intervention (e-BI) and 7 were controls. Seven had a parent or guardian present during the screening and intervention conducted within the trial. As with the parent trials, interviewees from the low risk trial tended to be younger than those from the high risk trial. Among female interviewees, those from the high risk trial tended to have been allocated to the face to face intervention and those from the low risk trial had been allocated to the control or e-BI conditions.

### Materials

To guide the interviews a semi-structured topic guide (see S1 File) was developed which explored young peoples’ views about the research, screening and intervention processes. This guide predominantly focused on the acceptability of methods but was flexible, permitting the addition of issues emerging from earlier interviews and allowing participants to raise any issues they felt were important. The topic guide was not piloted but was revised following the completion of the first seven interviews. Some closed questions were amended to more open phrasing but no further changes were made.

### Transcription and analysis

Length of interview varied from just 5 minutes to 45 minutes with the majority lasting between 10 and 25 minutes. All interviews were audio-recorded and transcribed verbatim before being anonymised. To minimise burden on participants’ transcripts were not returned to them for comment. Framework analysis [43], an approach recommended for qualitative health research [44], was employed. An initial framework for coding based on participants’ experience and understanding of the different stages of the research process (approach, screening, intervention and follow up) was developed and left flexible enough to accommodate additional issues emerging from the data. Initial application of this framework identified a number of emergent themes relating autonomy and beneficence with further ethical considerations emerging in codes relating to each stage of the research process. This led the research team to employ the four guiding principles of biomedical ethics to structure coding, data analysis and interpretation, and provide an overarching framework for discussing the findings. Three researchers (EL, CE, MB) independently read transcripts and coded data within this framework using NVivo 10. Researchers and two senior staff (RMcG, EK) discussed codes on an on-going basis with emergent themes added to the framework and any disagreement in interpretations resolved. The initial descriptive account of the data was refined through further group discussions, leading to the final interpretation. Findings are supported by exemplar quotes (interviewees identified by gender, age, trial and intervention). Data saturation was considered to have been met when the first twenty interviews had been complete with no new themes or contradictory responses emerging, however recruitment continued to 27 participants to ensure diversity across the purposive sampling criteria.

### Ethics and Governance

The studies were granted ethical approval by the National Research Ethics Service London—Fulham (ref:14/LO/0721). The trial registration reference was: ISRCTN45300218 dated 5th July 2014.

## Results

### Autonomy

The involvement of young people in research is in itself an acknowledgement of their autonomy. Many of the young people in this study voiced support for youth participation in research that was relevant to them. Some also explained the added benefit of engaging young people in the co-production of materials to ensure that they were appropriate and appealing to the target group:

*I think it’s a good idea to ask like the younger ones of what they would think would be best to pass on*, *more information to younger ones rather than asking like adults*.

(Female/17/High-Risk/face-to-face)

*Yeah I think so*, *I think they need to sort of be more involved and make it easier to understand for them because it sort of applies more to people their age*.

(Male/17/Low-Risk/E-BI)

However, autonomy encompasses much more than just supporting the idea that an individual has something to offer in terms of research data. In order to be autonomous and provide informed consent young people must feel at ease about being approached, have a clear understanding of what participation will involve, what their rights are as a participant and what they are being asked to do. Young people in this study were happy to be approached whilst in emergency care and some thought it was a good place to capture a diverse sample:

*I think it's a good way of like getting a good sample of people I guess*.

(Male/16/High-Risk/E-BI)

*I felt it was fine*, *I wasn't fazed by it at all*

(Male/14/Low-Risk/Control)

Nevertheless, for some, the issue of being approached may not have been fully considered until the interview.

**I**: *OK*, *and what did you think about being asked to be involved in a study about alcohol whilst you were in A&E*?

**P**: *Erm*, *I didn’t really think about that*.

**I**: *Yeah*.

**P**: *It didn’t really cross my mind*

(Female/16/High-risk/face-to-face)

Adolescents reported understanding their rights as participants with specific references to confidentiality, right to refuse and right to withdraw. Much of this understanding appeared to be gained from the verbal explanations of the trials provided by research staff rather than the written information sheets that were given to participants:

*Well she showed me what her like research like what it was the project was about* [*mmhhmm] and she explained that if I don’t want to do it then it’s totally up to me like and everything’s confidential and I was totally agreed with her and I just said I would do it for her no bother and I just did it for her*

(Female/14/Low-Risk/Control)

It was evident that participants were clear that the decision to participate was their own and that they could have time to consider their participation. There was no evidence in the interview transcripts that participants felt they should seek approval or guidance from parents or guardians when deciding whether to participate and no suggestion that they felt ill-equipped to make the decision alone:

*she came to me holding my name*, *and was very pleasant and made it very clear from the start*. *She gave me a few minutes to sort of have a think about it*… *and I came back to her and agreed to take part*. *And then filled out all the information*, *and yeah she was nice and friendly*, *and very approachable so yeah*

(Male/17/High Risk/Control)

Participants also identified that the research itself had been clearly described, or ‘explained rather well’. In support of this they were also able to offer descriptions of the research study which broadly fitted its purpose and hence showed some understanding of the aims of the project and what participation would involve:

*If I took part it would like help you get a better understanding of how it could pass information to younger people about the causes of drink and that*.

(Female/17/High-Risk/Face-to-face)

However, none of the descriptions demonstrated a full understanding of the randomisation process or the differences between trial arms. Instead participants often spoke about participating in a *‘survey’* and seemed more focused on topic than study design:

*I just thought it was a survey to ask about like young peoples’ lifestyles and what they do*.

(Female/17/High Risk/Face-to-face)

Similarly, participant descriptions of the research tended to focus on completion of the baseline measures and the initial trial visit with limited detail pertaining to follow up visits being offered even when specifically asked about this aspect of the trial:

*I can't remember I think she might have put my details down*…*she said you'll probably get a letter through the post and I got that last week and then obviously I had the phone call yesterday*

(Female/14/Low-Risk/Control)

Because participants were interviewed up to a year after being enrolled in the trials, recall inaccuracy may have contributed to misunderstanding. Some of the participants voiced this issue in interviews.

*Oh god I can’t remember*, *erm ah it’s a long time ago*.

(Male/14/Low-Risk/Control)

When asked during the qualitative interviews, participants could not think of any aspect of the approach or explanation of the research which could be improved. Nevertheless, it is clear that care needs to be taken when communicating the complex aspects of research design.

Participants identified that they had understood the screening questions; however, a small number of participants suggested that some questions could have been clearer. These participants reported seeking clarification from trial staff who were then able to provide the required assistance and enable continued participation.

*Some of the questions were a bit erm confusing let’s say*, *I mean I wasn’t completely thrown by it but some of them you did have to think about*.

(Male/17/High-Risk/Control)

Regarding the interventions specifically, the majority of participants were happy to receive information and advice about alcohol; with no suggestion of difficulties in understanding the advice provided.

*I was given a leaflet and she explained the leaflet as well*… *I understood her*, *I understood what she was saying*

(female/17/High-Risk/Face-to-Face)

Information was predominantly considered to be relevant, appropriate for the age group and some participants recognised the non-judgemental approach to delivery:

**P**: *I was aware of the risks but it did highlight other key things that I wasn’t aware of*

**INT**: *do you think that the information or how it’s delivered could be improved at all*?

**P**: *no*, *no I think that was pretty good as well like she was just really nice about it like she didn’t make me feel like I’d done anything wrong or anything*

(Female/17/High-Risk/Face-to-Face)

### Beneficence

Most participants expressed clear views about the importance of research being conducted with young people, particularly when the research subject was relevant to them; they often mentioned the benefits for others:

*I think it helps not just themselves but everybody else out there*. *Just because we need to learn don’t we*

(Female/14/Low-Risk/Control)

Aside from a sense of altruism, some felt that participation in research was itself a positive experience:

*It was quite nice to be involved in the study*. *I thought it was quite interesting*, *yeah*.

(Male/16/High-Risk/E-BI)

With regards to the intervention, participants identified the availability and wide spread use of alcohol as a key reason for needing access to reliable and accurate information about alcohol. Further, participants recognised that being under the legal drinking age (18 years of age in the UK) does not protect one from this exposure to alcohol and thus younger adolescents should be included in interventions:

*Because they’re underage and they’re like exposed to alcohol you know…Most people at that age actually drink alcohol a fair bit* … *Yeah*, *it’s a good idea to involve under-ages*.

(Male/17/High-Risk/Control)

One participant also identified the issue of screening and intervening before potentially problematic behaviours develop, highlighting the benefits of universal and targeted approaches:

*We need to learn young before we get older and just think it’s acceptable*.

(Female/14/Low-Risk/Control)

Participants who received an intervention described the process as ‘informative’, ‘relevant’, ‘good’, ‘helpful’, or ‘useful’. Many felt they had gained additional knowledge about alcohol, such as learning how many units were in a particular drink:

*yeah it had some different things on like unit levels and things like that*, *stuff like that*, *that aren’t really taught*… *I was aware of the risks but it did highlight other key things that I wasn't aware of*

(Female/17/High-Risk/Face-to-face)

However, some participants described the content of interventions as already familiar to them from their parents and school lessons, with no additional knowledge or benefit having been gained with one stating:

*alcohol erm like I know everything about it I think even though I don*’*t drink it*

(Male/14/Low-Risk/E-BI)

Despite this, the advice was typically seen as helpful to participants and could have wider reaching harm reduction effects with friends and peers also potentially benefiting:

*if you are like having a drink and that and something does go wrong*, *what*, *what you should do*… *‘cos I know loads of people who don’t actually have a clue and say someone like is absolutely mortal on the floor they actually just leave them because they don’t know what to do*.

(Male/17/High-Risk/Face-to-face)

### Non-maleficence

When conducting research in healthcare settings one of the primary concerns of research staff is often ensuring that the research does not detract from or delay the care participants receive. No participants reported feeling they had experienced harm from participating in this research nor did they feel that it had influence their care:

*it seemed totally harmless and you know I was happy to do it…I'm totally open to it*.

(Male/16/High-Risk/E-BI)

*It didn't prolong me or delay me in any way and so I thought that was alright*.

(Male/17/High-Risk/Control)

Conversely, participation was seen to have the benefit of providing young people with something to do while waiting to be treated, something which did not necessarily extend to completion of the follow up sessions when some participants explained that they had other priorities:

*when you sort of see someone in hospital*, *you know approach them*, *you haven't really got*, *what I mean I don't want to offend but you know that's all they've got to do really*, *sitting in the waiting room*. *But when you follow them up*, *I think a lot of people don't really want to give you their time*

(Male/16/High-Risk/E-BI)

There was some evidence that those attending with alcohol-related injuries may have felt less comfortable taking part and that this may have resulted in socially desirable responses:

*I actually fell down the stairs the night before because I had alcohol…I didn’t tell the researcher at the time because it was very bad*.

(Female/17/High-Risk/Face-to-face)

With regard to potential harm from participation the manner and approach adopted by research staff including interventionists appeared to be important. Participants’ responses to staff approach and intervention delivery were positive with exchanges described as ‘nice’, ‘lovely’ ‘positive’ and ‘very welcoming’:

*she was just really nice*, *she just asked us nicely if I wanted to take part and I didn*'*t mind*

(Female/17/High-Risk/Face-to-face)

Beyond this young people were primarily concerned with confidentiality. Alcohol use can be a sensitive subject with scope for embarrassment. Most participants, whether from high or low risk categories, reported that screening did not present a problem for them as long as confidentiality and privacy were assured:

*I guess because all of the information is like private and everything you could erm*, *it is sort of erm*, *what's the word*? *Acceptable*. *Obviously if it wasn't kept secret and some information was leaked it could affect that person's*, *say*, *chances of getting a job or something*. *But I guess as it's a confidential study then it's alright*, *it's acceptable*.

(Male/17/High-Risk/Control)

The importance of ensuring confidentiality is evidenced by the fact that some participants sought assurance from the researchers that their responses would be protected:

*“I did ask her at the time and she said no one would know*”

(Female/17/High-Risk/Face-to-Face)

Within this, a sub-theme of ‘parental presence’ also emerged from the data. When a parent is present during research, screening or intervention procedures the circle of confidentiality may be expanded to include not only the participant and the researcher but also the parent. In this work, there was no requirement for parents to be present during completion of the baseline measures or intervention delivery. Instead, participants attending the ED with a parent or guardian were offered the option of moving away from their parent or guardian during participation. Some accepted this offer while others declined. This potentially reflects the finding that opinion was split with regards to the acceptability of having a parent present during screening or intervention. Some described being ‘*absolutely fine*’ with having a parent present, while others explained they would ‘*prefer to do it without her [mother] there’*. Although the majority of our participants reported feeling comfortable discussing alcohol use in front of a parent or guardian, some still expressed concern for others:

*Some people [who] might not want their parents to know about that sort of thing but I’m not particularly fussed*.

(Male/16/High-Risk/E-BI)

There was also evidence that the presence of a parent or guardian could inhibit participants’ ability to talk freely or accurately about their alcohol use:

*say if somebody like my parents were there*, *I would say that I don’t drink at all*. *But when they’re not there*, *I can be more honest so I think*, *what was it*, *a private interview or something*

(Female/17/High-Risk/Face-to-face)

Completing the screening questions on an electronic device was seen to offer a greater sense of confidentiality and allow participants to protect their answers even in the presence of a parent:

*no one can see what you’re doing*, *which is pretty good*… *it was a bit personal if someone didn’t want to*, *you know*, *especially with your mum there*

(Male/17/High-Risk/Control)

Finally, participants considered, not only their own experiences but also those of others who might be more vulnerable than they perceived themselves to be, highlighting the importance of having appropriate and carefully considered inclusion criteria:

*Because my injury was not like fatal*, *and I was alright*, *I was OK*, *it would be OK for me*, *but I think somebody was in a lot of pain and they're waiting for emergency*, *like serious*, *serious*, *then for somebody to approach them about something completely unrelated could be annoying to them and they might get angry*.

(Female/17/High Risk/Face-to-face)

### Justice

Participants did not report feeling ‘singled out’ and were aware that researchers were approaching all individuals within the age category for inclusion with one participant going so far as to identify the questions as ‘standard practice’:

*Yeah*, *I mean it didn't really worry me at all*, *I wasn't thinking they were going to attribute something to me because I've got a broken leg*, *so*. *But no*, *it didn't feel like they came to me for a particular reason*, *I think it was just like a random sample*, *wasn't it*? *people between certain ages like*, *yeah*

(Male/17/High-Risk/Control)

*the questions didn’t*. *you know*. *they didn’t like offend or upset me [mmm] they just seemed like standard practice so that was fine*.

(Male/16/High-Risk/E-BI)

The issue of facilitating widespread participation seemed to be of importance to many interviewees. In this case the intervention was seen to be useful and accessible to the target population though potentially of more use in the future:

*I'll probably use it more in the future when I'm older and I drink more often*… *it was really good for the age category that it's aimed at 'cos there's not too much information that you get bored of reading it but there's enough so that you know exactly the importance of alcohol*.

(Female/17/High Risk/E-BI)

Again, the importance of involving adolescents, in the co-production of materials was identified as potentially enabling effective communication with this age group:

*Yeah I think so*, *I think they need to sort of be more involved and make it easier to understand for them*

(Male/17/Low-Risk/E-BI)

Further to this, the use of technology was predominantly seen as helpful for those who had difficulties with reading or writing but participants identified that this would possibly have the opposite effect for research with older populations who were considered to be less familiar with technology:

*people like me who've got dyslexia*, *it's probably a bit messy writing it down by hand so by doing it on the iPad it's a lot quicker and neater*, *so yeah*.

(Male/17/High Risk/Control)

*it depends on who your target audience is*, *because obviously I’m young*, *and young people wouldn’t mind but if you were targeting a more older audience like people with diabetes and stuff and older people*, *they might sort of like*, *not know how to use the technology and stuff*

(Female/14/Low-Risk/Control)

While young people generally saw the benefits of technology in widening participation there did appear to be some difficulties relating to accessing the e-BI intervention. Specifically, one participant had not downloaded the app and another had deleted it due to not having sufficient memory space on their phones, while one more explained that the app was not available in the app store so they had never downloaded it.

## Discussion

In this study, many of the interviewees recognised the importance of young people having the opportunity to take part in research on topics of significance to them. The findings generally support the acceptability of alcohol screening, interventions and alcohol intervention research with adolescent populations in emergency care. We found no indication that alcohol intervention per se or the emergency care setting was viewed as unacceptable to participants.

However, acceptability was dependent on certain criteria being met. Firstly, the friendly, non-judgemental approach adopted by research staff appeared to be important and is something that should be maintained in future research and intervention work. Secondly, confidentiality must be assured. Some participants pointed to the benefit of completing questions on an ipad or tablet in affording them a greater sense of privacy and reducing concerns about the potential for their responses to be misplaced. In some cases maintaining confidentiality also meant having the option to complete screening and receive intervention without a parent present. Although the presence of parents or carers during consent and/or intervention activity seemed to be accepted by many participants, some were less comfortable about parental presence and felt this might inhibit their own and others’ ability to speak freely. This could, in turn, limit identification of those who would benefit most from intervention delivery or identification of the most appropriate intervention to deliver. This finding supports previous research which reported that parental involvement could restrict adolescent uptake of healthcare [45] especially among those engaged in risky behaviours [46]. Future research should carefully weigh the benefits of having parents present during adolescent research participation against the potential for gaining more honest and open responses if participation is completed one to one. Although there was no evidence in this work that participants felt the need to defer to or consult their parents when making decisions about research participation consideration should also be given to how best offer participants a legitimate choice to complete research activities in private if they are attending treatment with a parent. Finally, it was important that young people were aware that they were not the only ones being approached and thus that they did not feel ‘singled out’. This has direct implications for future intervention work: although targeted interventions allow the delivery of the most relevant information a universal approach to screening and identification is likely to be more acceptable to young people.

Most of the adolescents we interviewed appeared to have a good understanding of their rights as participants, including the fact that participation was voluntary, and of many aspects of the trials procedures, particularly the subject matter, this is in line with previous research [47–49]. However, specifics relating to the technical design of the trials including randomisation procedures and follow up were less well reported. This may simply be a result of the time which elapsed between initial participation and interview, alternatively it may be an indication of limited understanding of these details. Although shortfalls in understanding are not uncommon in research with adult participants [50] this finding demonstrates the importance of providing information in a clear, succinct manner, and offering opportunities for participants to seek clarification to inform their decision making. In this work, much of the understanding of the research and research involvement appeared to be gained from the verbal explanations offered by staff rather than the written information sheets that participants were given. As such allocating additional time for researchers to verbally introduce research and discuss participation may enhance understanding more than provision of additional or alternative written guidance.

Similarly, where participants had difficulty understanding questions in the baseline questionnaire, this was overcome by seeking clarification from the researcher highlighting the importance of having researchers available to provide assistance and guidance if needed. Many young people in this work pointed to the benefits of involving young people in the co-production of research and intervention materials to ensure their acceptability to the target audience. Indeed, the co-produced intervention materials employed in this work were generally found to be appropriate for adolescent participants. As such, involving young people in the co-production of baseline questionnaires may help to overcome difficulties in understanding and interpretation but this can be problematic when existing validated tools, especially those that do not have child or adolescent specific variants available, are utilised. Finally, with poor recollection relating to follow up, using initial contact by text message, email or postal mail to prompt recall of study participation may facilitate completion of follow up visits.

In line with previous research, many of the young people also appeared able to assess possible implications of research participation and weigh up decisions about participating [51], based not only on relevance and helpfulness to themselves but also to other people [52], typically younger adolescents. The benefits identified included research participation itself as well as knowledge gained from the study interventions—generally seen as interesting, relevant and helpful to participants, who welcomed having something to do while waiting for treatment in the emergency department.

There was no evidence to suggest that participants experienced any harm as a result of involvement or that talking about alcohol with adolescents would lead to adverse consequence such as encouraging initiation of drinking or increased consumption. Over half of our participants were reportedly drinking at risky levels whilst the remainder had not really started to consume alcohol. Nevertheless, they all described alcohol as a normative behaviour—a view supported by other work (e.g.[12 53]). Many individuals in the low-risk trial described the intervention content as useful for ‘when’ (not ‘if’) they started drinking alcohol. That alcohol consumption was already framed as inevitable highlights the need for effective interventions to reduce future health risks.

Although some participant responses were more succinct than others this was considered to be typical of the way young adolescents speak and the assumption that a young person who talks less during an interview has provided less useful data has been queried [54]. These accounts not only appeared authentic but they also provide some reassurance about the possibility of social desirability during interviews. The primary limitation of this work is that participants who agreed to be interviewed had already participated in the trials and may be more positive about the issues being explored or better informed about the topic than those who elected not to take part. This may in part account for why participants offered few criticisms of the trial or areas for improvement. Where those who decline initial participation are comfortable giving reasons for non-participation, collection and analysis of this data could provide additional insight and areas for improvement, though it is important for ethical research conduct that individuals not be required to provide such information. In this work, although purposive sampling was adopted, participants also self-selected in that they had to be contactable at the time of the interviews and had to agree to take part. Diversity was achieved across age, gender, high or low risk status and allocated intervention however, the final sample had limited diversity with relation to participant ethnicity (80% of participants identified as ‘white’). Even taking into account the majority white participant pool in the parent trials non-white participants are still under represented in this sample. Further to this, a high number (n = 71) of those contacted did not respond to contact. This may be symptomatic of the minimal number of attempts made to contact each participant about the study (n = 3). While this helped reduce any pressure participants may have felt to participate it also means that only those who were easy to reach participated. The fact that interviews took place around a year after initial participation in the trial, something which likely contributed to limited recall of certain aspects of the research and potentially contributed to shorter, less detailed responses. A final limitation to consider related to the framework analysis employed. While the principles of biomedical ethics were adopted as an overarching structure based on the themes emerging from the data and all major codes were able to be captured within this framework an alternative approach to coding may have led to the identification of different themes.

## Conclusions

The research and intervention methods were generally found to be acceptable. The perceived relevance of the study seemed to be a key influence on willingness to become involved. The universal approach to screening, assurances of confidentiality and the non-judgemental approach of researchers contributed to acceptability which may in turn be inhibited by parental presence. Typical adolescents in this study appeared to understand the implications of participating in research; they described a process of considering potential benefits and harms both for themselves and for other people during the consent processes. Nevertheless, it is clear that many of the adolescents in this study did not have a full understanding of the specific research design. Future work would benefit from engaging young people in identifying how to explain the technical aspects of research designs as well as in the co-production of study materials and processes.

## Supporting information

S1 File Appendix interview topic guide. (DOCX)

S2 File Appendix excerpts from interview transcripts. (DOCX)

S3 File COREQ checklist. (DOCX)

## Abbreviations

AUDIT: Alcohol Use Disorders Identification Tool
AUDIT-C: Alcohol Use Disorders Identification Tool Consumption
e-BI: Electronic Brief Intervention
