# Supplementary material for: Adolescent perspectives about their participation in alcohol intervention research in emergency care: A qualitative exploration using ethical principles as an analytical framework
Source: PLoS One. 2019 Jun 12;14(6):e0217855. doi: 10.1371/journal.pone.0217855 (PMC6561559; doi:10.1371/journal.pone.0217855)
Supplement: S1 File — (DOCX) [file pone.0217855.s001.docx]

**Topic Guide for Interviews with Young People**

**Introduction:**

Thank you for taking part in our study about young people and alcohol. I have some questions I want to ask you about your involvement in the study to help us understand how best to talk to young people your age about alcohol.

Explain confidentiality

Explain voluntary nature of participation

Ask permission to tape record – explain how data will be used.

Any questions?

Complete consent form

**Consent**

1. Before you agreed to take part in the study, what was your understanding of what it would involve?
2. How clearly did the researcher explain the study?

Prompts:

Understanding that participation was voluntary

Understanding that participation was unrelated to care

Understanding of what study would involve for them

1. What did you think about being asked to be involved in a study about alcohol whilst you attended ED?

Prompts:

How did the researcher approach you?

Where you were approached – waiting room or private space

1. Is there anything you think we should do differently when explaining the study to young people and asking them to take part?

**Screening**

1. You were asked questions about your alcohol use, what do you think about being asked these questions?

Prompts:

Acceptability of questions

Acceptability of iPad

Acceptability of venue when completing

Was parent present/nearby

Burden of time

Impact upon care

1. How did you find answering the questions on the iPad?

Prompts:

Understanding of questions

Acceptability of questions

Acceptability of venue when completing

Was parent present/nearby

Burden of time

Impact upon care

1. Is there anything we should do differently when asking young people to answer questions on the iPad?

**Intervention (arm 2 only)**

1. What were your thoughts on the alcohol advice the researcher gave you?

Prompts:

Acceptability of level of information

Acceptability of the way it was delivered

Acceptability of venue

Was parent present/nearby

Burden of time

Impact upon care

1. What did you know about alcohol before you were given this advice?

Prompts:

Knowledge of units

Knowledge of risks

How knowledge differed to advice given

1. How could this information be improved?

**Intervention (arm 3 only)**

1. How did you find using the SIPS City street app?

Prompts:

Were they able to download

Ease of use

Ease of navigation

Incentive/motivation to use repeatedly

1. What do you think about the information in the app?

Prompts:

Acceptability of the information

Level of information

Relevance of information

Graphics

Language

1. What did you know about alcohol before you used this app?

Prompts:

Knowledge of units

Knowledge of risks

How knowledge differed to advice given

1. How could the app be improved?

**General and follow up**

1. Do you think that it is important for young people to be involved in research?
2. As part of the study, we are following up young people 6 months from when they answered questions in the ED, were you expecting to be followed up?

Prompts:

How did you think you’d be contacted

Were you happy to be contacted

Could this have been made clearer

1. Are there difficulties involved in following up young people as part of a study?

Prompts:

Preferred method of contact: e-mail/telephone (call or text)/ postal

1. What do you think the challenges of involving young people in research are?
2. Is there anything you would like to add about your involvement in this study?
