# Supplementary material for: Adolescent perspectives about their participation in alcohol intervention research in emergency care: A qualitative exploration using ethical principles as an analytical framework
Source: PLoS One. 2019 Jun 12;14(6):e0217855. doi: 10.1371/journal.pone.0217855 (PMC6561559; doi:10.1371/journal.pone.0217855)
Supplement: S2 File — (DOCX) [file pone.0217855.s002.docx]

**Excerpts from Interview Transcripts**

This document provides data excerpts and additional quotations from the interview transcripts. These are grouped within the coding framework. The initial framework for coding was based on participants’ experience and understanding of the different stages of the research process (approach, screening, intervention and follow up) and was developed and left flexible enough to accommodate additional issues emerging from the data. Initial application of this framework identified a number of emergent themes relating autonomy and beneficence with further ethical considerations emerging in codes relating to each stage of the research process. This led the research team to employ the four guiding principles of biomedical ethics to structure coding, data analysis and interpretation, and provide an overarching framework for discussing the finding. As such the data below are presented under the overarching themes of ‘Autonomy’, Beneficence, ‘Justice’ and ‘Non-maleficence’. These are then divided into sub-themes which either emerge from coding (e.g. ‘young people in research’) or relate to the phases of research involvement (e.g. ‘Follow-up).

| **Autonomy** | | | | |
| --- | --- | --- | --- | --- |
|  | | **Young People in Research** | | |
|  | |  | “INT: ok, and do you think that it’s important for young people to be involved in research?  YP: yeah like I think it helps not just themselves but everybody else out there”  P: Erm, I don’t know, just to help out I guess, you know. And you know, from my wisdom, like if someone never tells you anything then you will never know about certain things, like alcohol, so yeah, I think it’s just, I don’t know [laughs].  “INT: do you think it’s important for young people to be involved in research?  I: yeah I think it’s a good thing so I do”  “YP: err yeah because there’s more young people than …. not necessarily the younger generation [UNCLEAR] it can affect young people as they’re growing and it can affect their body more [unclear]”  “INT: Cool. And do you think it’s important for young people, or people aged between fourteen and seventeen to be involved in alcohol research?  P: Yeah.  INT: Can you think why it might be important?  P: Because they’re underage and they’re like exposed to alcohol you know.  INT: Exactly.  P: Most people at that age actually drink alcohol a fair bit.  INT: Yeah, exactly. And so you think it’s a good idea?  P: Yeah, it’s a good idea to involve underages.“  “INT: very few last questions, I know you said you don’t mind taking part in surveys, do you think it’s important for researchers to involve young people in research?  YP: yeah I would say so because there is like loads of young people not aware of alcohol and do need to sort of sometimes like get just get told about it.”  “YP: I think it’s a good idea to ask like the younger ones of what they would think would be best to pass on more information to younger ones rather than asking like adults.”  “YP: Yeah I think so, I think they need to sort of be more involved and make it easier to understand for them because it sort of applies more to people their age.”  “INT: Alright. So the last few questions are do you think it’s important for young people to be involved in research like this?  P: Yeah yeah.  INT: And why do you think it’s important?  P: Just because we need to learn don’t we.  INT: Yeah exactly.  P: We need to learn young before we get older and just think it’s acceptable.”  “YP: yeah everybody should be in research because it’s health and safety anyways but and just everyone takes alcohol, everyone might die and we won’t do anything and we can’t do anything about it (mhmm) it’s just a mistake, something wrong that everyone does unfortunately.“  “INT: OK. And so just coming to the final questions, do you think it’s important for young people to be involved in research?  P: Yeah.”  “INT: do you think it’s important that we involve people in this type of research?  YP: Yes.  INT: And I know this is quite unexpected, but why why do you think it’s important that we do that?  YP: Because it’s the truth, like it’s normal people, like yeah.”  INT: and what did you think about being asked to be involved in a study about alcohol?  YP: ahhh haven’t, at the age, like 16 it’s something that obviously sort of comes into people lives and it’s just sort of it’s probably sensible you know I don’t really mind. “  “INT: and some general questions, and I’ve only got 2 or 3 more. Erm, do you think it’s important for young people to be involved in research?  YP: er yeah yeah I think I think you know with the topic of alcohol, it is really important for people you know (mhmm) sort of learn more things sorry yeah”  “INT: and erm you know so you were asked questions about alcohol use…?  YP: aha  INT: what did you think about being asked those types of questions?  YP: erm I thought I thought it was really good because people our age often go out every Friday and Saturday night and just drink  INT: mhmm  YP: until like 12’o clock  INT: ok mhmm so it’s sort of relevant to what lots of your friends are doing?  YP: yeah”  “INT: mhmm and, erm so I mean in general, do you think it’s important for young people to be involved in research?  I: yeah”  “YP: erm it’s good to in touch with the young people because young people often do silly things when they’re teenagers and all that and they don’t often think about what they’re doing.  INT: so I mean do you think that just sort of being involved in research makes people think a bit more?  YP: yeah” | |
|  | | **Consent** | | |
|  | |  | **ED Context** | |
|  | |  |  | “INT: Were you happy being approached there?  YP: Aye, yeah”  “INT: Were you happy being approached there?  YP: erm yeah it was quite good I was just… because I wasn’t really expecting it I was just in A&E ‘cos I had hurt my finger (mhmm) and then it was yeah it was fine at all.”  “INT: Were you happy being approached there?  YP: yeah I was totally fine with that it was just a couple of times when I was waiting in the waiting room it just took the time”  INT: OK, OK and what did you think about being asked to be involved in a study about alcohol  YP: I felt fine, I wasn’t fazed by it at all it’s erm… yeah.  INT: and in terms of, you know obviously you were waiting in A&E at the time, was that OK being approached there?  YP: yeah it was fine”  “INT: mhmm erm and were you ok so were you approached in the waiting room?  YP: yeah I was yeah I was in erm I was in a room with my dad yeah  INT: mhmm and was that was that sort of acceptable to be approached there?  YP: yeah yeah yeah I mean it was a hospital yeah it was totally fine.”  “YP: erm not really no it was probably the only time that you’d be able to catch them you know I didn’t personally I didn’t have a problem so so yeah it was ok “  “INT: what do you think about being asked to be involved in a study about alcohol when you were attending A&E?  YP: ah, it was alright”  “INT: ok and was that just in the waiting area at the hospital?  YP: yeah  INT: and were you happy to be approached like that?  YP: yeah”  “INT: And what did you think about being asked to be involved in a study about alcohol while you were in A&E  YP: I was happy to do it like, because I don’t like I wasn’t in there for alcohol reasons”  “INT: ok and were you happy with the way that you were approached?  YP: yeah”  INT: So what did you think about being asked to be involved in a study about alcohol whilst you were at accident and emergency?  YP: I didn’t really mind. I was happy to answer the questions.”  “INT: Great. So you didn’t mind being approached whilst you were at accident and emergency?  YP: No it was OK.  INT: OK. Do you think it could’ve been done better in any way?  YP: I don’t really have any complaints about it, it was fine.”  “YP: Yeah I think it’s a good way of like getting a good sample of people I guess. “  “INT: And what did you think about being asked to be involved in a study about alcohol whilst you were in the A&E?  YP: Erm I don’t know, I was fine with it.”  “YP: I was comfortable with it, not a problem.”  “INT: Excellent. And so what did you think about being asked to be involved in a study about alcohol whilst you were in accident and emergency for another reason? Do you know what I mean?  YP: Yeah, I mean it didn’t really worry me at all, I wasn’t thinking they were going to attribute something to me because I’ve got a broken leg, so. But no, it didn’t feel like they came to me for a particular reason, I think it was just like a random sample, wasn’t it, people between certain ages like, yeah.”  INT: So the next question is what did you think about being invited to take part in a study on alcohol? Like, did it concern you? What did you think, while you were attending the emergency department, and somebody actually came up to and asked you to take part in a study, did you expect that to happen?  YP: Er, no.  INT: OK, and did you find being invited to take part in the study as something quite unexpected?  YP: No.  INT: It was alright?  YP: Yeah it was.“  “YP: No, it was quite ironic because I actually fell down the stairs the night before because I had alcohol”  “YP: It was very ironic. I didn’t tell the researcher at the time because it was very bad and so the reason I said I was at the hospital was not for the reason I was saying but the real reason was that. It was ironic when I was doing it, but it was fine, it was OK [laughs].”  “YP: Because my injury was not like fatal, and I was alright, I was OK, it would be OK for me, but I think if somebody was in a lot of pain and they’re waiting for emergency, like serious serious, then for somebody to approach them about something completely unrelated could be annoying to them and they might get angry and stuff but that’s because I was free, if I was in like a lot of pain and or blah blah blah then maybe I wouldn’t even want to do it, do you know what I mean? I think it depends on each individual.”  “INT: Great. And what did you think about being asked to be involved in a study about alcohol while you were in the accident and emergency department?  YP: Er, it was fine.  INT: OK, erm, and where were you approached initially, you know, by the researcher?  YP: I was waiting in the A&E.”  “INT: In your experience, do you think there’s anything we could be doing differently?  YP: No, I thought it was all fine.”  “INT: And what did you think about being asked such questions in an accident and emergency department?  YP: I think it was alright er for like the age group I was in. “  “INT: (laughs) Yeah. And where did the researcher approach you, was it in the waiting room or?  YP: I was in the waiting room and they come up to me.  INT: Was it unexpected?  YP: Yeah yeah.  INT: But you didn’t mind?  YP: I didn’t mind, no.”  “YP: I wasn’t like that bothered about it and it was probably a good idea to help with other people who were like having problems with alcohol and things.”  “INT: OK, and what did you think about being asked to be involved in a study about alcohol whilst you were in A&E?  YP: Erm, I didn’t really think about that.  INT: Yeah.  YP: It didn’t really cross my mind”  “INT: And do you remember whether that was somewhere private, or was it in the waiting room?  YP: It was, I think it was, somewhere private.  INT: Yeah. And do you think that whole process of being approached, do you think we could’ve done it better in any way?  YP: No, I don’t think so.”  “YP: She came out and she explained what the research was about and asked if I was willing to take part and then took us into a different room and got us to fill in my detail and answer the questions.  INT: So the study that was initially explained when you were in the waiting room?  YP: Yeah  INT: and was that ok – I mean was that an ok place to be approached?  YP: yeah that was fine.  INT: and then the room you went into – was that ok as well?  YP: yeah that was fine”  “INT: mhmm and do you think that it was ok to be asked, while you were in A&E, to be involved in a study?  YP: yeah, I think it’s alright yeah”  “INT: mhmm  YP: don’t see why it wouldn’t be, it’s the same as walking up to someone in the street really isn’t it?”  “INT: I just wondered what you thought about being asked to be involved in a study about alcohol when you were in A&E?  YP: yeah.  INT: erm I mean was that ok to be asked those sorts of questions?  YP: yeah yeah fine.”  “YP: Yeah it was fine, because with it being on an iPad, like nobody really knew what was going on “  “INT: ok, and what do you think about being asked to be involved in a study about alcohol whilst you were in A&E? Were you happy with the way that you were approached?  YP: yeah because I was just say there waiting to go in and he just came over and he asked quite politely if I could do a survey.”  “INT: ok, er and I mean what do you think about being asked to be involved in a study about alcohol and lifestyle when you were in A&E?  YP: It didn’t bother me at all, I’d gladly take part”  “INT: mhmm and were you, was it the waiting room where you were approached?  YP: yeah  INT: and was that ok? were you happy to be approached in that place?  YP: yeah it was alright, fine.”  “YP: Yeah, I was like on a bed and they literally just came [laughs].”  “INT: Cool, thanks. Erm, so in terms of us asking you to be in the study, do you think there’s anything we could be doing differently when we explain to people about the study?  YP: No, I think it’s literally well done explanation-wise. No, I think it’s OK actually. “ |
|  | |  | **Ethics & Rights** | |
|  | |  |  | “YP: well she showed me what her like research like what it was the project was about (mmhhmm) and she explained that if I don’t want to do it then it’s totally up to me like and everything’s confidential and I was totally agreed with her and I just said I would do it for her no bother and I just did it for her”  “INT: And did you know that it was entirely voluntary?  YP: yes  INT: and were you told that it wasn’t related to the care that you would receive at the hospital  YP: yeah”  “YP: Oh, I think it was a survey about teen alcoholism or you know and like to find out just general health and safety about it and things like that just a general enquiry and it was confidential.”  “INT: mhmm and did you sort of understand that you didn’t have to take part if you didn’t want to?  YP: yeah yeah yeah it was totally optional  INT: and you knew it wasn’t related to the care that you’d receive at the hospital?  YP: yeah”  “INT: ok, and did you know what, I mean did you know what your actual involvement would be, that you’d be answering questions about those things [yeah] and … did you think that the researcher explained the study well to you?  YP: yeah”  “INT: Did you know that you didn’t have to take part if you didn’t want to?  YP: yeah  INT: and that it wasn’t related to any of the treatment that you received at A&E  YP: yeah”  “INT: and did you feel like taking part was voluntary?  YP: yeah  INT: so did you feel like you could have said no if you wanted to?  YP: yeah  INT: and did you think that it was related to your care or treatment at the hospital?  YP: pardon?  INT: did you feel like it was related to your care or treatment at the hospital?  P: like nah, I just thought it was like a random questionnaire thing.”  “INT: OK. And then how clearly did the researcher explain the study to you? Like did you understand it was voluntary?  YP: Yeah yeah yeah I understood.  INT: And that it was unrelated to the treatment that you were going to be receiving?  YP: Yeah.  INT: Yeah, and did you also know that you could say no, that you didn’t want to take part if you didn’t want to?  YP: Yeah I knew that.  INT: OK good. And so you knew it wasn’t related to your treatment, so you didn’t feel pressure to take part?  YP: No no, it was entirely voluntary. “  “INT: Yeah. And so did you understand that it was completely voluntary for instance?  YP: Yeah I understood that.  INT: And that it was unrelated to your care? You know you went to accident and emergency for another reason.  YP: Yeah.”  “YP: Yes, everything was made clear to me. All the ethical considerations were made clear to me, that I could pull out at any point.”  “INT: Lovely, thanks. And did you think it was related to your care or treatment that you were going to receive in accident and emergency?  YP: I don’t think it was sort of relevant particularly because I had a trauma injury so erm and it was my understanding that the survey was on the amount of alcohol consumed within a couple of months so it wasn’t really relevant to that extent, but yeah. “  “INT: Excellent. And did you realise that it was completely voluntary?  YP: Yes, yeah.  INT: Yeah and that it didn’t relate to your care in the A&E, you know did you understand that he or she wasn’t a doctor or a nurse?  YP: Yeah yeah.”  “YP: Yeah, and I was asked if I wanted to do it and I said yes.”  INT: Excellent. And how clearly did the researcher actually explain the study to you? Did you understand that it was completely voluntary, and that you didn’t have take part?  YP: Yeah.  INT: You understood that?  YP: Yeah.  INT: Did you also understand that it wasn’t related to the care that you would receive in accident and emergency?  YP: Yeah.”  “INT: OK, cool, and did you understand that it was completely voluntary and that you didn’t have to take part in the research?  YP: Yeah.  INT: Or did you feel pressured?  YP: No, it was fine.  INT: OK, and did you know that the research was completely unrelated to the care that you would receive?  YP: Yes.”  “INT: I mean, for instance, did you understand that it was completely voluntary on your part, and that you’d be doing us a favour by taking part?  YP: Yeah.  INT: And did you understand also that it was unrelated to your care that you would receive at the hospital?  YP: Er yeah.  INT: Yeah er and so all of that was clear?  YP: Yeah.”  “INT: Good one. Erm, I mean did you understand that it was all voluntary, like you were doing us a favour?  YP: Yeah yeah.  INT: And that you didn’t have to take part?  YP: No no, I knew I didn’t have to take part.  INT: Excellent. And did you understand that it was unrelated to your injury or accident?  YP: Yeah yeah.“  “INT: And did you understand that it was entirely voluntary?  YP: Yeah.  INT: And did you understand that it was unrelated to the treatment that you were going to be receiving?  YP: Yeah.  INT: And so you knew it wasn’t related to the care in A&E that you were going to be receiving, that it was something separate?  YP: Yeah.  INT: Yep, good.  YP: Yeah, I did.”  “INT: mhmm and did you understand that it was an optional thing to take part….  YP: Yeah  INT:…and that it was unrelated to the care that you’d receive at the hospital?  YP: yeah”  “INT: do you remember being clear that it wasn’t compulsory  YP: yeah yeah I knew it was like voluntary…  INT: and that it wasn’t related to your hospital treatment?  YP: yeah”  “INT: and did you feel like taking part was voluntary?  YP: yeah I didn’t feel like I had to  INT: and could you have said no if you wanted?  YP: yeah yeah that was fine  INT: and did you think that it was related to your care or treatment at all?  YP: erm no”  “INT: And I mean did you know that you didn’t have to take part..  YP: yeah  INT: and it wasn’t related to the care that you’d receive at the hospital?  YP: yeah I know “  “INT: and the other thing was: did you understand that it was not related to your medical care?  YP: yeah I did  INT: and that it was voluntary so you didn’t have to take part if you didn’t want to?  YP: yeah I understood that.”  “YP: erm no, I think it was alright because I got, like I got asked if I wanted to and I got explained it was all voluntary and what it was for.”  “INT: and did you feel like taking part was voluntary? That you could say no if you wanted to?  YP: oh yeah, he said any time you wanted to say, like if you wanted to stop he said just say stop you didn’t have to answer all the questions you were uncomfortable things like that  INT: and did you… was it clear that the questions you were asked wouldn’t be related to the care that you’d receive [erm yeah] at the hospital, so once they’d explained it to you did you feel like you had a clear idea about what it would involve?  YP: yeah”  “INT: and did you know that you didn’t have to take part if you didn’t want to?  YP: yeah  INT: and that it wasn’t related to your care at the hospital?  YP: yeah”  “INT: So did you know that you didn’t have to take part?  P: Yeah yeah.  INT: And did you understand that it was unrelated to your care that you were going to be receiving in accident and emergency?  YP: Yeah yeah.” |
|  | |  | **Reason for being involved** | |
|  | |  |  | “YP: I thought it was a good way to pass the time as I was there”  “We were in the waiting room, there’s nothing much to do so…”  “YP: Yeah, yeah, yeah. I was basically just waiting to be called in but that obviously was going to take time so it was just something I didn’t mind doing because I was free I was just sitting there doing nothing so I might as well have done it.” |
|  | |  | **Understanding of the study** | |
|  | |  |  | “YP: No I totally understood it all, she was very welcoming, she was very like giving detail about what the project was about and she made us feel very positive about it all”  “INT: Thanks. Do you think there’s anything we should do differently when we explain this study to people when inviting them to take part? Can you remember anything that might improve how we can do that?  “YP: I think it was alright. I don’t think you need to improve anything.”  “YP: If I took part it would like help you get a better understanding of how it could pass information to younger people about the causes of drink and that.”  *“*YP: I just thought it was a survey to ask about like young peoples’ lifestyles and what they do.“  “YP: It was about like, like your lifestyle and like exercise and stuff like that"  “YP: She said to me that it was about alcoholism but I don't remember right"  *“*YP: I can't remember I think she might have put my details down…she said you'll probably get a letter through the post and I got that last week and then obviously I had the phone call yesterday”  “YP: She told me like what the aim was, like what was going to happen, and why it's happening"  “YP: it was quite clear like I knew what I was getting involved"  “YP: it was 100% clearly like I knew what I needed to do and she explained that it was just for a study, everything like that" |
|  | |  | **Approach** | |
|  | |  |  | “INT: OK. And so were you happy with how you were approached?  YP: Yeah it was alright.  INT: Yeah cool and the way they were speaking with you, was that acceptable too, was it alright?  YP: Yeah there was nothing wrong with that at all.”  “YP: Erm, she she, I think she had a list of people and their names, and obviously between the ages, and obviously she came to me holding my name, and was very pleasant and made it very clear from the start. She gave me a few minutes to sort of have a think about it… and I came back to her and agreed to take part. And then filled out all the information, and yeah she was nice and friendly, and very approachable so yeah.”  “YP: yeah that was fine it was really nice the lady was really lovely”  “YP: I was just…because I wasn't really expecting it, I was just in A&E 'cos I had hurt my finger (mhmm) and then it was fine"  “YP: erm no she was just really nice, she just asked us nicely if I wanted to take part and I didn’t mind so…”  "YP: The girl was really nice when she asked me the questions and stuff" |
|  | | **Screening** | | |
|  | |  | “YP: Erm I guess, some of the, on the actual iPad, some of the questions were a bit erm confusing let’s say, I mean I wasn’t completely thrown by it but some of them you did have to think about, and I guess that if a few questions were a bit clearer then people could’ve given a more accurate more accurate information and data”  “YP: It was only on a couple of questions which I was confused. The others I knew”  “YP: I understand them, like I understood them clearly"  “YP: erm I think I asked her on a couple of them, just what the word meant because I didn’t understand how they were worded in the questions (ok) so it was the rest of them were fine it was just a couple of ones where I didn’t understand ‘cos I get like I always struggle when the way questions are worded”  “YP: P: Yeah yeah I understood. I didn’t understand a few that I had to ask about. Yeah, got there eventually”  “YP: I had like a few problems with some questions but I just asked the guy that was there"  *“*YP: Yes, really easy to understand, yeah" | |
|  | | **Intervention** | | |
|  | |  | “YP: I was given a leaflet and she explained the leaflet as well… I understood her, I understood what she was saying”  “YP: I think it's quite good because it's educational and it informs people of the alcohol and how alcohol misuse can affect the body"  “INT: do you think that the information or how it’s delivered could be improved at all?  YP: no, no I think that was pretty good as well like she was just really nice about it like she didn’t make me feel like I’d done anything wrong or anything”  “YP: I didn't mind, at first I didn't know if it was 'cos like I though they thought there was a problem with what I had said you know what I mean [OK] like with how I had answered it but yeah I didn't mind being given them at all" | |
|  | | **Follow-up** | | |
|  | |  | “INT: were you expecting to be followed up  YP: erm I was just yeah I told her, she asked us if I would like to be like a part of it and I said yeah and I can’t remember I think she might have put my details down  INT: ok  YP: and then she said you’ll probably get a letter through the post and I got that last week and then obviously I had the phone call yesterday and the phone call today and then I’ve had another one now.  INT: ok, so you weren’t surprised when you got when you got that call asking you to do a few more questions?  YP: No, like I was like ready for it ‘cos I knew I was going to get the call”  “INT: and as part of the study we’re following up young people 6 months from when we first asked them the questionnaires, I’m not sure if it’s been 6 months yet since you answered the questionnaires but were you expecting to be followed up?  YP: yeah yeah I was, I think I was in April”  “INT: mhmm and did you recently get a phone call, sorry I think you already said you got a phone call recently to answer a few more questions?  YP: yes  INT: and were you expecting that phone call I mean did you know you were going to be followed up in 6 months’ time?  YP: Yes” | |
|  | | **Poor recall** | | |
|  | |  | “INT: Good, cool. Erm, and so do you remember receiving some knowledge or advice on alcohol by any chance?  YP: Erm, yes, I think so. I can’t really remember what”  “YP: I didn't know, I couldn't remember, I can't remember what I was told"  “INT: Yeah, it’s alright. Did you receive an app for your phone or did you have a conversation with the researcher or, did anything like that occur?  YP: Erm, I vaguely remember him mentioning an app I think, I don’t know. He just spoke to me about it.  INT: And were you shown an app, do you remember seeing it?  YP: I can’t remember.”  “YP: Oh god I can’t remember, erm ah it’s a long time ago.”  “INT: OK. So we’re nearly finished. I was just wondering, do you remember receiving an intervention?  YP: No.  INT: Did someone go through a, did they give you like a five minute chat about alcohol? Do you remember?  YP: Oh yeah yeah, I remember.” | |
| **Beneficence** | | | | |
|  | | **Overall** | | |
|  | |  | “INT: ok, and do you think that it’s important for young people to be involved in research?  YP: yeah like I think it helps not just themselves but everybody else out there”  INT: Can you think why it might be important?  YP: Because they’re underage and they’re like exposed to alcohol you know.  INT: Exactly.  YP: Most people at that age actually drink alcohol a fair bit.  INT: Yeah, exactly. And so you think it’s a good idea?  YP: Yeah, it’s a good idea to involve underages.“  “INT: And why do you think it’s important?  YP: Just because we need to learn don’t we.  INT: Yeah exactly.  YP: We need to learn young before we get older and just think it’s acceptable.”  INT: and what did you think about being asked to be involved in a study about alcohol?  YP: ahhh haven’t, at the age, like 16 it’s something that obviously sort of comes into people lives and it’s just sort of it’s probably sensible you know I don’t really mind. “  “INT: and some general questions, and I’ve only got 2 or 3 more. Erm, do you think it’s important for young people to be involved in research?  YP: er yeah yeah I think I think you know with the topic of alcohol, it is really important for people you know (mhmm) sort of learn more things sorry yeah”  “YP: no not really, I mean I’m totally open to it and you know it’s I’ve got a voucher out of it so I’m totally fine. “  “YP: I thought it was a good way to pass the time as I was there”  “YP: We were in the waiting room, there’s nothing much to do so…”  “INT: and erm you know so you were asked questions about alcohol use…?  YP: aha  INT: what did you think about being asked those types of questions?  YP: erm I thought I thought it was really good because people our age often go out every Friday and Saturday night and just drink  INT: mhmm  YP: until like 12’o clock  INT: ok mhmm so it’s sort of relevant to what lots of your friends are doing?  YP: yeah”  “INT: mhmm and, erm so I mean in general, do you think it’s important for young people to be involved in research?  I: yeah”  “YP: erm it’s good to in touch with the young people because young people often do silly things when they’re teenagers and all that and they don’t often think about what they’re doing.  INT: so I mean do you think that just sort of being involved in research makes people think a bit more?  YP: yeah”  “INT: And when you think about how it took to complete the iPad study, did you think it was a bit burdensome, or was it quick, or was it long, what did you think about the time it took?  YP: It was the right amount of time. It took basically as long as long as for like what I needed for, because I was waiting after having been seen like to get a follow-up appointment date, and that’s the time it took me to do it.“  “YP: It was quite nice to be involved in the study. I thought it was quite interesting, yeah.”  “YP: I was waiting for ages man.  INT: Yeah.  YP: It helped.  INT: It helped pass the time?  YP: Yeah”  “YP: Yeah, yeah, yeah. I was basically just waiting to be called in but that obviously was going to take time so it was just something I didn’t mind doing because I was free I was just sitting there doing nothing so I might as well have done it. “  “YP: Erm, er, what would it be? No, she was really good, she was. She gave me a prize, like five pounds, maybe give me like ten next time. [Both laugh] Make the prize higher.”  “YP: It was a bit of a pass-time, so it was OK.”  “YP: I wasn’t like that bothered about it and it was probably a good idea to help with other people who were like having problems with alcohol and things.”  “INT: and did you feel like doing the questionnaires influences your treatment at all? I mean even if that was being made to wait longer  YP: ahh no, not at all, it helped pass the time sort of thing” | |
|  | | **Screening** | | |
|  | |  | “INT: and what did you think about the ipad? I mean how did you find answering the questions on it?  YP: yeah it was easy, like obviously some places you get you have to like write it with a pen and it’s more like it felt more confidential because the paper could go missing whereas like on the system on the ipad I felt more safe about it all.”  “YP: yes, I thought it was a good way to use it to put the questions across.“  “INT: right, and, I think the last one in this section, is just is there anything at all that you could do differently when we’re asking people to answer questions on the iPad?  I: no, I thought it was, went down very well.”  “INT: ok and in terms of how usable the ipad was, was that an ok way of delivering the questionnaire?  YP: er yeah yeah totally knew how to use it so it wasn’t an issue at all”  “YP: the iPad was good because on pieces of paper they could get ripped and all that but with the iPad you can it’s just a straight up answer and all that so…”  “INT: was that better than something else like a pen and paper  P: yeah so much easier”  “INT: Thanks. And what did you think about answering questions on the iPad?  YP: I thought it was quite an interesting way of doing a questionnaire, and I think it worked fine.”  “INT: Alright, thanks. And going back to the iPad, how did you find that, was it alright answering questions on an iPad rather than, say, like a piece of paper or something?  P: I’d prefer the iPad, I’d say.”  “YP: Yeah yeah, I mean, you’ve got the technology there, which makes it quicker, and erm makes it quicker and more accurate I guess. You don’t have people like me [laughs] who’ve got dyslexia to erm like erm, sorry I’ll just shut the door, yeah so people like me who’ve got dyslexia it’s probably a bit messy writing it down by hand so by doing it on the iPad it’s a lot quicker and neater, so yeah.”  “YP: Yeah, so it’s OK, it was good. Much better than writing and everything like, it’s better to click on things, it’s easier.”  “INT: Yeah, and what did you think about answering questions about alcohol on the iPad?  YP: That was fine.  INT: Yep, and would you prefer that to the face-to-face encounter?  P: Yes.“  “INT: Erm, yeah, and what did you think about erm being given an iPad to answer questions about alcohol in the assessment unit? Did you think that was acceptable?  P: Yes.”  “INT: OK. And how did you find using the iPad?  YP: I found that it was an easy and better way of doing it because like it was more interactive than just like writing down or ticking a box like on a piece of paper or something.  INT: So you thought it was a better method than like, say, using a pen and paper?  YP: Yeah.”  “YP: that was better because it didn’t mean you were doing loads of written answers.”  “I: ok, and what did you think about the actual using the iPad itself, was that ok?  P: yeah yeah it was fine like it was pretty clear there were no like problems.”  “INT: and how do you find using the iPad  I: oh yeah it was really easy and it was nice and it was better than doing it on a piece of paper or like on a form it didn’t feel too formal”  “INT: and you were asked questions using the iPad, what did you think about that process?  I: ahh it’s like a new generation process and it makes it easy to answer the question very fast “  “YP: Yeah it was fine, because with it being on an iPad, like nobody really knew what was going on“  “INT: Yeah and I mean I suppose compared to other ways of asking questions like on paper….  YP: Yeah I think that’s a better way to use it”  “INT: mhmm and how was the experience of using the iPad  P: that made it a lot easier than through paper and things like that”  “YP: Yeah, it seemed like a good machine, like it’s really good, and no one can see what you’re doing, which is pretty good.”  “INT: OK. And did you think it was OK answering questions about alcohol on the iPad, rather than, say, talking to a human? Was it alright?  YP: Yeah, it was better, because it was like a bit personal if someone didn’t want to, you know, especially with your mum there.”  INT: Yeah.  YP: That way you could just keep it to yourself, you know, it’s kind of confidential as well. “ | |
|  | | **Intervention** | | |
|  | |  | “INT: uhhuh and in terms of what sort of information it was, was that information that you knew already or was any of it …  YP: aye, aye I knew it all aye, most of it  INT: So there wasn’t anything new there?  YP: ah, not really, I’ve already done most of it at school”  “INT: ok and I mean in terms of your knowledge of alcohol, did you feel like that was, you know when somebody went through the app with you that you gained any knowledge about alcohol?  P: yeah I think I did, like different units and how much you actually take  INT: ok and was that something that you weren’t aware of before?  P: yeah like I didn’t know”  “INT: Aha. And so did you already know about alcohol before?  P: Yeah, not as much.  INT: Not as much. Erm, so did you think the advice was useful?  P: Yeah it was.“  “INT: And did you know anything about alcohol before taking part in the study?  YP: Yes.  INT: You did, OK. Did you have any knowledge of what a unit of alcohol might be?  YP: Sorry?  INT: Did you have any knowledge, any idea, of what a unit of alcohol might be?  YP: Yes, yes.  INT: And you understood about the risks?  YP: Yes.  INT: OK. And do you think there’s anything that could’ve been improved about any information that we gave you about alcohol?  YP: No, I think it was alright actually.”  “INT: Did you think that the advice that the researcher gave you, did you think that that was informative?  YP: It was informative, yeah.  INT: And did they deliver it well to you, could you understand?  YP: Yeah, they did, yeah, they did.”  “YP: Yeah, they did, and it was very usual that they had visuals as well like, they had, they had a sheet, a leaflet or something, which actually had the pictures on it and everything. They were just talking through it but it was all there, it was quite, it was quite good.”  “YP: Average, yeah I knew average, like well basically alcohol is not good for you anyway, regardless of how much you’re drinking, it’s just not good for you, and when you drink it you already know so. But yeah, I didn’t know like, you know when they say a pint, like I didn’t know how many pints are OK, even though it’s not OK at all but how many pints is OK. But then again everybody has a different endurance level, so yeah.”  “INT: And were you aware of the risks around alcohol as well?  YP: What do you mean?  INT: Like just some of the effects and risks of drinking alcohol, before being in the study?  YP: Oh yeah yeah, yeah I knew that injury and falling over and all that sort of stuff.”  “INT: And what did you know about alcohol before, before the research? I mean, did you have an adequate amount of knowledge about the risks and  P: Yes I did.  INT: Cool. And did you receive that from, well I suppose it’s advertised, but do you find out about alcohol through family, through friends, through school?  P: Yes.  INT: Yeah, so everything that we mentioned was already familiar to you?  P: Yes.”  “YP: Good  INT: You found it good, can you say a bit more?  YP: Er, erm, I don’t know, er, I just think it was helpful.  INT: Helpful, yeah yeah.  YP: Like it tells you how to like consume it, like how much.”  “INT: OK, so I know you said it was helpful, but would you say it enhanced your knowledge of alcohol from what you previously knew?  P: Yes.  INT: It did.  P: Yeah.”  “INT: Did you have any knowledge beforehand about alcohol?  P: Yeah yeah, I knew about what it does and that.”  “INT: what did you think our advice was like? How did it compare to what you already knew? Did you know it all anyway?  P: Yeah yeah I knew it all anyhow. “  “INT: And did you know much about alcohol before all of this?  YP: Yeah.  INT: You did? Did you know about what a unit of alcohol was, by any chance?  YP: I don’t think so.  INT: Did you know like the risks of alcohol?  YP: Yeah.  INT: And the knowledge that we gave you about alcohol, was it different to knowledge that you’d been told previously?  YP: I think some of it probably was.  INT: Did the information we gave you coincide or conflict with information we gave you?  YP: I think it went with it.“  “YP: I think it’s quite good because it’s educational and it informs people of the alcohol and how alcohol misuse can affect the body”  “INT: was there any way that the information that could be changed to make it more relevant?  YP: Just information about binge drinking and how that affects youth’s bodies because they’re not as adapted to… older [mhmm]”  “INT: And in terms of what you knew about alcohol before using the app, do you think you had an understanding of alcohol and the risks?  YP: not massively, I mean obviously I understood that it could affect your liver and stuff but I didn’t have a full understanding of it”  INT: ok – so did you know about units of alcohol?  YP: not [erm] I hadn’t really, paid attention before.  INT: OK and is that something that you found out during use of the app?  YP: yeah  INT: so in terms of how your own knowledge differed from advice given in the app, was there any differences?  YP: no not really  INT: And if you were, if you were redesigning the app, is there any way that you could think, I mean any way at all that it could be improved?  YP: Just like I said add some more information about binge drinking and how it affects the young body and how it can leave permanent damage.  INT: ok, so quite specific information about that [yeah’ and that would be in terms of how it affects different parts of the body or brain?  YP: yeah both.”  “YP: I think my general knowledge has improved (mhmm) but sort of like you know with your parents like ah you shouldn’t do this you shouldn’t do that so it was relatively good you know. “  “YP: yeah it had some different things on like unit levels and things like that like stuff like that that aren’t really taught if you know what I mean [yeah] like I didn’t I didn’t know that yeah  INT: ok and in terms of, there was some more information about risks, was that something that you were aware of or was that new as well?  YP: I was aware of the risks but it did highlight other key things that I wasn’t aware of”  “INT: I mean did you think you had… what sort of knowledge do you feel you have about alcohol?  YP: alcohol erm like I know everything about it I think even though I don’t drink it  INT: ok…  YP: how does it affect the human and that stuff.  INT: mhmm  YP: yeah”  “YP: not personally, ‘cos like I know like the common sense bit of alcohol.  INT: mhmmm so there wasn’t anything new there that you were given?  YP: ah no  INT: and do you think that we could give out any other information that would be more useful I mean to yourself? Is there anything that we could have covered that we didn’t?  YP: err err phooo err I can’t remember if it was on like that but say if you are like having a drink and that and something does go wrong, what, what you should do  INT: ok so some sort of practical advice?  YP: yeah ‘cos I know loads of people who don’t actually have a clue and say someone like is absolutely mortal on the floor they actually just leave them because they don’t know what to do.  INT: ok, so sort of talk about some of the issues involved when people are drinking?  YP: yeah”  “YP: Just that it was helpful as well and when I got the leaflet that was helpful because, as I said before, it give you information about who you could talk to, where you could go and like what units and that was in drinks.  INT: okay so the units bit, the how much….  YP: And there was a one for stopping smoking as well, where you could go  INT: okay, so that bit and the contact details was helpful  YP: yeah”  “INT: mhmm and was there anything that you found out from the leaflet?  YP: erm not really because I’d like I already knew quite a bit, with me dad.  INT: ok, so you’d had a conversation with your dad?  YP: no not really, he’s an alcoholic and I’d been speaking to me family about it [ok] and I knew part of the dangers about it and that”  “YP: I didn’t know too much about it sort of it was just yeah nah didn’t really know much about it I just knew that it was alcohol and it makes you drunk really.”  “INT: mhmm…. And did you, did you have knowledge about alcohol before being given that information?  YP: yeah I knew a little bit of information about [UNCLEAR] alcohol  INT: I mean I guess what I was wondering is if any of the information given to you was new information?  YP: some of it was yeah  INT: and, do you think it could be improved at all? I mean is there information about alcohol that you would like but I didn’t get?  YP: no, it was alright I was more aware of what it was now apart from ….[UNCLEAR]” | |
|  | | **Poor recall** | | |
|  | |  | “INT: Good, cool. Erm, and so do you remember receiving some knowledge or advice on alcohol by any chance?  YP: Erm, yes, I think so. I can’t really remember what.”  “INT: Yeah, it’s alright. Did you receive an app for your phone or did you have a conversation with the researcher or, did anything like that occur?  YP: Erm, I vaguely remember him mentioning an app I think, I don’t know. He just spoke to me about it.  INT: And were you shown an app, do you remember seeing it?  YP: I can’t remember.” | |
|  | **Justice** | | | |
|  | | **Approach** | | |
|  | |  | “INT: my next questions was; what were your thoughts on being asked to take part in a study about alcohol when you attended the A&E?  YP: I thought that it was a good idea that they were approaching everybody in the age category.”  “YP: But no, it didn’t feel like they came to me for a particular reason, I think it was just like a random sample, wasn’t it, people between certain ages like, yeah.”  “YP: Yeah, it was random I think, but the study was about alcohol and young people, so I was picked I think, there were lots of different people there, but I think the fact that I was picked was random. “ | |
|  | | **Intervention** | | |
|  | |  | “INT: ok, and I was going to ask how, how was downloading the app, is that something you’ve downloaded since?  YP: I don’t think I have  INT: ok, so other than the, you were shown it in the actual session but you haven’t used it since, is that…  YP: yeah  INT: ok, so I mean I was going to ask, I mean was that because you weren’t interested in, I was just interested in, was there a reason why you didn’t download the app afterwards?  YP: it was just, I don’t know, I was at was at low risk anyway [ok] so I didn’t really see why  INT: ok, so you didn’t think that the information would be that relevant to you?  P: yeah”  “INT: Am I right that after you, after you finished the questionnaire you were shown a phone app?  YP: erm, yeah it didn’t really work though, I think they tried yeah it didn’t work.”  “INT: and then did they sort of give you some information about downloading that if you wanted to afterwards?  YP: yes I think yes  INT: was that something you went on to do?  YP: No it wasn’t erm I I tried and the app, the app wasn’t actually available on the app store when I tried.  INT: Oh, ok, you did attempt to (yep) and you weren’t able to ok. OK”  “YP: Oh yeah. Yeah yeah, there was a leaflet.  INT: Or you might’ve received an application for your smartphone?  YP: Oh yeah yeah I did, but I forgot about it.  INT: Can I just ask you, did you download it?  YP: No I forgot.”  “YP: I didn’t get an app or anything, but they did tell me about like erm, portions of alcohol like, I asked because like it’s all drinking shots and stuff but I didn’t “  “INT: Yeah they did? Or did you receive an application for your smartphone?  YP: Yeah they told me about the application, yeah.”  “INT: OK, did you by any chance download the application?  YP: I think my mum might’ve downloaded it when we were there.  INT: Yeah. Did you have a look at it?  YP: Yeah.  INT: Cool. It’s just if you can think back, did you think it was easy to download the application?  YP: Yeah.  INT: And have you used it more than once?  YP: I don’t think so.”  “INT: OK. And do you remember what it was like to use, like moving stuff around and navigating?  YP: Yeah, it was like really easy.  INT: It was easy, yeah. Did you read any of the information that was included in the app?  YP: Yeah.  INT: And what did you think about the information? Like, was it too simple, what were the graphics like, was the language too complex?  YP: No I think it was quite good and informative.  INT: And do you think it was at the right level for you, you know it wasn’t too simple?  YP: Yeah.”  “YP: I only used it once or twice [mhmm] and it was really easy to access”  “INT: And what about the app in terms of ease of use, how did you find using it?  YP: really easy it was quite it was really well set out for young people “  “YP: I’ll probably use it more in the future when I’m older and I drink more often.”  “YP: no, like I say I think it was really good for the age category that it’s aimed at ‘cos there’s not too much information that you get bored of reading it but there’s enough so that you know exactly the importance of alcohol.”  “INT: oh sorry. In terms of how the app looked you know [ahh yeah] the sort of the street layout and the going into different buildings, what do you think about the appearance of the app?  YP: I think it’s a really good idea, it’s better than opening an app to just find pages and pages of information, it’s better because it’s more appealing. You don’t actually feel like you’re taking information in, you just feel like you’re using an app.”  “INT: OK and in terms of the language that’s used in the app, do you have any thoughts on that?  YP: not really, it was quite easy to understand but other people might not feel the same.”  “INT: mhmm and how did you find using that?  YP: It was all pretty smooth, it was quite explanatory and it sort of, it just it works well. “  “INT: and did you go on to download that yourself?  YP: I did for a bit but I ended up needing the storage space on my phone so I had to delete it.”  “INT: and in terms of the relevance to you, was that information which was useful?  YP: err parts of it were but obviously you know like the alcohol thing, I don’t really drink that much so it didn’t really apply all that much to me.  INT: and in terms of the way that was presented, obviously in the study we were speaking to 14 year olds to 17 year olds so there’s quite a difference and did you think that the language that we used was ok for someone of your own age?  YP: yeah it was pretty easy to understand, all the words and stuff. “  “YP: I don’t really think so, ‘cos it does give quite a lot of information and I think it it, like it, I don’t know like, I don’t know how to explain it, it’s like it does give the information but it’s not too much to handle if you know what I mean.”  “INT: ok, and am I right in thinking when you got to the end of the questions you were shown an app on an iPad  P: yes yeah I was  INT: and how did you find using that?  P: erm I haven’t used it yet I think I don’t think” | |
|  | | **Follow-up** | | |
|  | |  | “INT: do you think there’s any difficulties in particular with trying to follow up young people, I mean sort of catching them on the telephone, or e-mailing, or writing letters, I mean do you have any thoughts on that?  YP: What do you mean like…  INT: I suppose so, what’s the best way, to follow up, to contact people?  YP: erm probably on the telephone ‘cos all teenagers these days are always on the phone there’s never a minute when it’s not in their hand or near them.”  “INT: is there, are you sort of wary of answering the phone to people who you don’t know?  YP: erm like yesterday when they first rang I was like ‘mam should I answer it?’ but then she had erm recalled that the letter that I’d got they said I would get a phone call  INT: ok  YP: so I was like right I’ll answer it and then obviously I spoke to you and then..”  “YP: erm everyone has their phone on them a lot of the time, you know you caught me and I was able to sort answer straight away, I think post is a bit more difficult than contacting someone by mobile, but I think they’re all sort of good methods, just because erm I think post sometimes people just sort of see letters and think ahh well just bin it or something and they wouldn’t respond.”  “YP: yeah I sort of saw 0191 and I though oh I’ll pick it up, I don’t know who it is and I thought if it was a marketing I’d just sort of hang it up. So that is an issue yeah I think maybe I don’t know e-mails are quite good I think because they’re accessible and you know who they’re from.”  “INT: and was that, was that, were you happy to be called?  YP: erm yeah  INT: and do you think there’s any difficulties involved in following up young people?  YP: uhuh.  INT: like if you see an unknown number on your phone are you wary of it?  YP: It’s like I often answer it to see who it is and then if I’m not sure who it is I often hang up but in this case it’s the hospital so I just kept on the phone.”  “YP: err obviously you know 6 months is quite a while so people might like change their numbers and stuff so that could be a bit of a difficulty, especially with young people because they’re quite unaware, like they’re quite forgetful so might not be update their information to you per se.  INT: ok that’s interesting and I mean do you think lots of people are likely to change their phone number?  YP:it depends really, on sort of you know but it is quite a common occurrence isn’t it so it could happen at any time really it’s just sort of luck of the draw.”  “YP: yeah yeah mobile phone’s ok, fast and easy.  INT: mhmm and do you normally I mean would you normally answer your mobile if you didn’t know the number?  YP: yeah yeah yeah I do.”  “YP: I think mobile is better because if I see an email I’d just probably ignore it.” | |
|  | **Non-maleficence** | | | |
|  | | **Overall** | | |
|  | |  | “YP: yeah yeah it was totally fine you know it seemed totally harmless and you know I was happy to do it.”  “YP: I actually fell down the stairs the night before because I had alcohol…I didn’t tell the researcher at the time because it was very bad”  “YP: I'm totally open to it and you know it's I've got a voucher out of it so I'm totally fine" | |
|  | | **Impact on care** | | |
|  | |  | “INT: I wondered if you felt like it delayed your treatment at all?  I: nah I was waiting to see a doctor  INT: ok so you weren’t doing anything else at the time?  I: no”  “INT: ok, ok so you didn’t feel like it got in the way of your treatment  YP: ahhh no no, it wasn’t it wasn’t a very major thing it was just ‘cos I thought I Had broken my finger because I like had an injury at school.  INT: ok  YP: it was just I was just in pain I just had to use my left hand to do it”  “INT: did that delay you at all in terms of when you did see the doctor?  YP: err no err I didn’t, when I was in I was in for something that needed to be seen immediately but still it didn’t delay me.”  “INT: and did you in terms of how much time it took, did it affect your treatment in hospital at all?  YP: erm no  INT: because you were just waiting was that?  YP: because I was just waiting to get referred onto a ward”  “YP: er about 15 minutes altogether ‘cos like we got interrupted by a nurse so about 15 minutes would have been alright.  INT: ok and was that, was the interruption becomes you were being called through?  YP: no they just had to take like a needle out of my hand”  “INT: and did you feel that doing the questions influenced your treatment in any way or maybe made you wait longer?  YP: no [ok] it was just while I was waiting anyway so…”  “INT: And when you think about how it took to complete the iPad study, did you think it was a bit burdensome, or was it quick, or was it long, what did you think about the time it took?  YP: It was the right amount of time. It took basically as long as long as for like what I needed for, because I was waiting after having been seen like to get a follow-up appointment date, and that’s the time it took me to do it.  INT: OK cool thanks. And did it interfere, did it impact on your treatment?  YP: No, not at all.”  “INT: Yeah. So did you feel like taking part in the study interfered or influenced your treatment in terms of it making you wait longer or anything like that?  YP: No, not at all. It was absolutely fine.”  “YP: Well I think some of the questions went round on themselves in particular I think some questions were asked twice or something so that might’ve prolonged the actual survey but I don’t think time was really an issue although I was waiting in A&E and so I did get called up just after I’d finished. I mean I think fifteen minutes was alright to be honest. It didn’t prolong me or delay me in any way and so I thought that was alright. “  “INT: Did the study interfere with it by any chance?  YP: I don’t think so, no.”  “INT: OK, it was alright. And did it interfere with your care at the accident and emergency department in any way?  YP: No, not at all, no.”  “YP: Yeah, it was OK. If the lady would’ve called me in, we would’ve stopped it anyway.”  “INT: And did it interfere in a negative way with your care, in any way?  YP: No, no.”  “INT: Cool. And did that whole process have any impact on your care that you received from the nurses and doctors?  YP: I don’t think so, no.”  “INT: OK, and did it interfere with or impact the care you received?  YP: No.”  “INT: And did it interfere with your care in the A&E department in any way?  YP: No no. “  “INT: Cool. And did it have any impact at all on your treatment? Like, for instance, did you have to wait longer?  YP: No.  INT: It was alright?  YP: Yeah.”  “INT: And I mean did you feel that affected your, the length of your wait in A&E or that affected anything else in relation to your care?  YP: no not at all”  “INT: mhmm and did you feel like the taking part in the study influenced your care at all? That might have been… did you have to wait any longer?  YP: no no not at all”  “INT: and did you feel like doing the questionnaires influences your treatment at all? I mean even if that was being made to wait longer  YP: ahh no, not at all, it helped pass the time sort of thing”  “INT: and did you feel that doing the questions influenced your treatment in any way? Did they perhaps make you wait longer?  YP: It didn’t make me wait longer, no, ‘cos there was some like a few people in front of us.”  “INT: OK cool thanks. And did it interfere, did it impact on your treatment.  YP: No, not at all.“ | |
|  | | **Confidentiality** | | |
|  | |  | “YP: It was a waiting room but it was er full of people and everyone was there. They didn’t tell me what the thing was about outside because obviously it might put off people so they said we want you take part in a survey, would you like to do it, and I was like yeah why not. Then we went inside and she explained what it was about and then I was like yeah I’ll do it, and then, yeah.”  “YP: erm yeah just like make it 100% clear that the answers are anonymous just so there’s no worry or anything  INT: ok, was that something that concerned you at the time?  YP: erm yeah but I did ask her at the time and she said that no one would know.”  “YP: No, not really, I didn’t mind doing it, nobody really knew what I was doing because I would have covered the thing.”  “INT: I mean it was private enough? Was it?  YP: yeah there wasn’t many people in?”  “INT: ok and where, can you remember where abouts you were when you completed the questionnaires?  YP: I was just in a little room  INT: ok, so that was sort of off from the main waiting room…  YP: yeah it was just a little place with like with little seats yeah  INT: ok and was somebody, was anybody with you when you did the questions?  YP: erm just the lady who asked us to do them”  “YP: I mean I guess as a young person it’s sort of alright now that we’re of the age to drink and that it’s almost like a social thing, I guess it’s alright for us to, within reason, because if we, not me in particular, say oh yeah we go out and get smashed every night it could sort of, I don’t know, I guess because all of the information is like private and everything you could erm, it is sort of erm, what’s the word, acceptable, obviously if it wasn’t kept secret and some information was leaked it could affect that person’s, say, chances of getting a job or something. But I guess as it’s a confidential study then it’s alright, it’s acceptable.”  “YP: Erm, basically, because it was like confidential and all, I didn’t mind. But it’s one of those things that if was done in front of loads of people, I wouldn’t be as comfortable in saying, say if somebody like my parents were there, I would say that I don’t drink at all. But when they’re not there, I can be more honest so I think, what was it, a private interview or something wasn’t it, or like, if it’s more of a public thing like when there’s too many people then the person who’s answering the question is going to feel intimidated and you can get a social desirability effect, and the Hawthorne effect, and all sorts so it’s better if, it’s better if the researcher, erm yeah, sort of gets the person on their own a little bit.” | |
|  | |  |  | **Parental Presence** |
|  | |  |  | “INT: were you attending A&E with a parent or a friend  YP: yeah yeah  INT: and were they nearby when you were answering them?  YP: yeah  INT: and I mean was that ok, sort of answering them in the presence of a parent?  YP: yep”  “INT: the other thing I was wondering was, were you at A&E with a family member  YP: yeah  INT: and when you answered the questions on the iPad were you then in a different place to your family member or were you in the same room?  YP: I was in the same room  INT: and I mean was that ok? Answering questions in front of somebody?  YP: it was completely fine”  “YP: yeah I was with my mam  INT: mhmm and was it ok being asked, I mean were you in the room with your mum when you were answering the questions or was she in a different room?  YP: erm yeah we were just in the waiting room and I just sat beside her, I think she moved a couple of seats down but my mam was totally fine with us doing it because she understood what it was for,  INT: ok, and were you happy to be answering those questions whilst your mum was there?  YP: yeah I was totally fine”  “ I: and actually, just related to that, were you in A&E by yourself or were you with anyone?  P: I was with my mum.  I: mhmm and when you answered the questions was that in her presence or was it in a separate room or how did that work  P: It was in a separate room there was just me and the err interview person.  I: ok yeah I was just going to ask if that sort of impacted if it was ok asking questions with your mum there but I guess she wasn’t around when you were actually asking questions.  P: erm no”  “INT: and did you say that your dad was with you in A&E?  YP: yeah  INT: and so when you were answering the questions, were you near him or were you in a separate room at that point?  YP: er I was in the same room as him yeah  INT: ok and was that was that ok  YP: yeah yeah yeah yeah”  “INT: OK and were you at A&E with your parents?  YP: yeah I was in A&E with me mam  INT: and so did you answer questions in front of her?  YP: yeah  INT: er and was that ok because they were about alcohol, so could your mum see the answers you were putting in?  YP: aha  INT: and was that, were you happy with that or would more privacy have been better?  YP: erm yeah often when you’re in front of parents you often hide stuff away because you don’t want them to know.  INT: yeah yeah and I mean did you feel that you couldn’t answer some things honestly?  YP: erm, I answered…. everything as honestly as I could”  “INT: mhmm and were you attending A&E with anybody  YP: erm yeah  INT: was that a family member? Or friend?  YP: my friend  INT: ok, and did they stay with you when you were answering the questions or did they [yeah] wait. OK and was that ok [yeah] you were happy to answer them in front of your friend?  YP: yeah”  “INT: And were you alright answering questions about alcohol when your parent was there?  YP: Yeah I was absolutely fine.”  “INT: OK. Erm and did you have a guardian nearby while you were doing it?  YP: Yeah she was in the waiting room.  INT: Yeah, and did that feel acceptable? To be completing questions about alcohol whilst you had someone like a guardian present, do you know what I mean?  YP: Yeah, nah that’s cool.”  “YP: Erm no, I did it in a room from the paediatrics area.  INT: Yeah, yeah, and did you feel comfortable doing it there?  YP: Yes, I was alone with the lady, it was alright.  INT: Yeah, and was a guardian or a parent close by?  YP: Yeah my mother, when I did the questionnaire, I was alone with the lady.”  “YP: I was with my mum when I attended A&E but it was just me and the researcher when I answered the questions.  INT: Ok, and was that ok? I mean were you happy to just you to go into the room?  YP: yeah it was fine.  INT: and, did you say it was your mum sorry [yeah] was your mum happy for you to go off and do them as well?  YP: yeah she was fine with them.”  “YP: I was with my mum.  INT: mhmm and when you answered the questions was that in her presence or was it in a separate room or how did that work?  YP: It was in a separate room there was just me and the err interview person.  INT: ok yeah I was just going to ask if that sort of impacted if it was ok asking questions with your mum there but I guess she wasn’t around when you were actually asking questions.  YP: erm no”  “INT: ok, and had you been, were you in, was someone attending A&E with you?  YP: yeah me mam was  INT: ok so you left her in the waiting room when you went into the other room~?  YP: yeah  INT: ok, and was that ok?  YP: yeah that was fine it was my choice just to let her wait  INT: and was your mum happy with that as well?  YP: yeah she didn’t mind”  “INT: and I mean you said that your mum was in the other room still [yeah] would that have been was that best that you did that by yourself that bit?  YP: yeah I’d prefer to do it without her there”  “INT: was there anyone else with you when you, were you attending A&E with somebody?  YP: yeah I was with my dad  INT: ok and I mean were you ok asking, sorry, answering questions on the iPad with him nearby?  YP: yeah”  “INT: the other thing I was wondering was, were you at A&E with a family member  YP: yeah  INT: and when you answered the questions on the iPad were you then in a different place to your family member or were you in the same room?  YP: I was in the same room  INT: and I mean was that ok? Answering questions in front of somebody?  YP: it was completely fine”  “INT: I wondered if you felt like it delayed your treatment at all?  YP: nah I was waiting to see a doctor  INT: ok so you weren’t doing anything else at the time  YP: no”  “INT: and were you at A&E by yourself or were you with a parent?  YP: I was with a parent yeah  INT: mhmm and was that, I mean was that ok being asked questions in front of them or were you in a separate place when you answered them?  YP: errr there was [UNCLEAR] I don’t know if anyone was in the waiting area and if I wanted there was a different room I think [UNCLEAR] but I was comfortable doing it there.”  “INT: OK and were you at A&E with your parents?  YP: yeah I was in A&E with me man  INT: and so did you answer questions in front of her?  YP: yeah  INT: er and was that ok because they were about alcohol, so could your mum see the answers you were putting in?  YP: aha  INT: and was that, were you happy with that or would more privacy have been better?  YP: erm yeah often when you’re in front of parents you often hide stuff away because you don’t want them to know.  INT: yeah yeah and I mean did you feel that you couldn’t answer some things honestly?  YP: erm, I answered…. everything as honestly as I could”  “INT: and was anyone with you when you answered the questions?  P: yeah me mam was  INT: ok and was that ok, I mean were you happy answering them in front of your mum?  P: yeah I was fine”  “INT: And were you alright answering questions about alcohol when your parent was there?  YP: Yeah I was absolutely fine.”  “INT: Cool, thanks. Do you think it might be a problem for some people?  YP: Yeah possibly for some people. Like some people might not want their parents to know about that sort of think but I’m not particularly fussed. “  “INT: And did you have a guardian nearby?  P: Yeah yeah.  INT: And how did that feel? Talking about alcohol when you had someone like next to you?  P: Oh I didn’t have the guardian next to me, it was confidential, I went in a room.  INT: Right, excellent.  P: I had my dad with me at the time.”  “YP: no I was with my dad at that.  INT: ok and were you ok answering questions in front of him?  YP: err yes, well he can’t speak English very well I’m his translator so…  INT: ok so it wouldn’t have made a difference whether he saw what you were writing or not?  YP: yeah yeah” |
|  | | **Screening** | | |
|  | |  | “INT: I suppose I was meaning, were you happy to be asked those types of questions?  YP: yeah I was yeah, totally fine yeah”  “YP: I thought it was fine, ‘cos I don’t drink alcohol, I’m quite young, I thought it was fine asking about it, I’ve got no problems about it.”  “YP: erm yeah I didn’t mind the questions, the questions didn’t you know they didn’t like you know didn’t offent or upset me (mhmm) they just seemed like standard practice so that was fine with answering them”  “INT: and erm you know so you were asked questions about alcohol use…?  YP: aha  INT: what did you think about being asked those types of questions?  YP: erm I thought I thought it was really good because people our age often go out every Friday and Saturday night and just drink  INT: mhmm  YP: until like 12’o clock  INT: ok mhmm so it’s sort of relevant to what lots of your friends are doing?  YP: yeah”  “INT: ok, just a few more questions if that’s ok? You were asked questions about your alcohol use, what do you think about being asked those sorts of questions?  YP: I wasn’t really bothered”  “YP: yeah I think they were fine they were all appropriate so….”  “INT: Thanks. So you know you were being asked questions about your alcohol use, what did you think about being asked those questions?  YP: I thought it was fair enough really.  INT: You thought it was acceptable?  YP: Yeah, I didn’t really have any problem with that.”  “YP: The questions? They seemed reasonable. I don’t remember them all exactly, but there was nothing wrong with them.  INT: There was nothing that stood out?  YP: No, not particularly.”  “INT: Yeah, being asked about your alcohol use.  YP: For me, it’s OK. Yeah, I find it easy, I mean it was OK.  INT: Did you find it acceptable that someone should ask you?  YP: Yes, yeah it was.”  “YP: No it was acceptable, yeah.”  “YP: No, it was comfortable.”  “INT: And what did you think about being asked those questions? Did you find it acceptable, for instance?  P: Yes.”  “INT: Alright, good one. And what did you think about filling out the questions on the iPad?  P: Yeah yeah, it was alright, just read the questions and answered them.”  “YP: Yeah, I thought it was alright, I didn’t really take it personally.”  “INT: So you were asked questions about your alcohol use, what did you think about being asked those sorts of questions?  YP: personally I don’t see a problem with it because obviously I don’t drink a lot, well I don’t drink really ever because y’kna, I’m 17…  INT: ok  YP:err but then there’s obviously some people might take it like personally if they’ve got stuff like that going on but personally no, I thought it was sound, not a problem”  “INT: and were you happy being asked those sorts of questions, you know the things that it was asking about  I: yeah I didn’t mind at all”  “INT: ok, and so you were asked questions about alcohol use and do you think, I mean are you happy to be asked about that?  YP: yeah, I’m happy.”  “INT: okay, so you were asked questions about your own alcohol use, what did you think about those questions?  YP: I thought they were fine, like, I dunno”  “INT: and in terms of the things they were asking, were those, were you happy being asked about those sorts of things  YP: yeah they weren’t exactly personal questions they were just questions you would ask basically”  “INT: ok, that’s great. And going on to the actual questions that you were asked. You were asked questions about alcohol use (yes) that was one of the things… what do you think about those sorts of questions? I mean are they ok? Are they…  YP:…yeah they’re completely fine, normal questions”  “INT: So you were asked questions about alcohol use, were you happy to be asked those sorts of questions?  YP: yeah it’s fine yeah”  “YP: Oh, it was OK, like it was fair enough questions, not too bad, like they were just normal questions.”  “INT: Did you think it was acceptable to be asked about your alcohol intake?  YP: Yeah I think it’s acceptable. I’m aware that many people do it, underage as well, so it’s not, you know, it’s not something we would hide or anything like that.” | |
|  | | **Intervention** | | |
|  | |  | “INT: do you think that the information or how it’s delivered could be improved at all?  YP: no, no I think that was pretty good as well like she was just really nice about it like she didn’t make me feel like I’d done anything wrong or anything “ | |
